# Chronic pain in refugee torture survivors

**DOI:** 10.7189/jogh.07.020303

**Published:** 2017-12

**Authors:** Gunisha Kaur

**Affiliations:** Weill Cornell Medical College, New York, New York, USA

The World Health Organization has declared the existing refugee migration an international humanitarian disaster, considering it the worst humanitarian crisis since the Second World War. The United Nations High Commissioner for Refugees in 2015 documented over 65.3 million forcibly displaced people worldwide, including more than 21 million refugees; this number will undoubtedly increase considerably given the acceleration of violent global conflicts and the resulting numbers of displaced people [1]. Despite a universal ban by the United Nations, 50% of all countries, including 79% of G-20 countries, practice systematic torture, affecting an estimated 5%-69% of refugees in the world [2]. Physicians in developed countries will typically encounter survivors of torture as refugees or asylum seekers [3]. Medical anthropologists have highlighted the importance of evaluating non-medical factors such as economic and social determinants of health, during the process of refugee assessment [4]. Experienced physicians in the field recommend taking a multimodal, interdisciplinary approach to refugee treatment, such as the use of alternate therapies including acupuncture and *t’ai chi* [5]. Yet the holistic paradigm of assessment and rehabilitation is incomplete without considering the potential of chronic, debilitating somatic pain experienced by survivors of torture.

Torture leads to a combination of physical and psychological trauma. In a systematic review of the prevalence of psychiatric conditions in refugee populations, Post Traumatic Stress Disorder (PTSD) was found in 30.6% and depression in 30.8% of individuals [6]. The literature on refugee survivors of torture has similarly demonstrated a high prevalence of chronic pain, with some data showing an incidence as high as 83% [7]. To describe this, our group conducted a retrospective, IRB approved study (N = 11) of an immigrant South Asian population in New York City using a novel pain assessment tool. Participants in the study were self-reported survivors of government-sponsored torture in Punjab, India. Torture was employed by police authorities in order to punish individuals, extract confessions, or obtain desired information. Most common mechanisms of physical torture identified included slapping, kicking, and punching, stretching of legs greater than 180 degrees, and muscle crush injuries, each with the persistence of severe chronic pain over two decades after torture. Constant, debilitating pain was described by all subjects, often related to specific mechanisms of torture and with no confounding medical condition to explain the disability (**Table 1**).

**Table 1:** Mechanism of torture, chronic somatic pain in subjects and whether or not this pain was related to torture mechanism.

Subject number	Chronic pain	Pain distribution related to torture
1	Yes	Yes
2	Yes	Uncertain
3	Yes	Yes
4	Yes	Yes
5	Yes	Yes
6	Yes	Yes
7	Yes	Yes
8	Yes	Uncertain
9	Yes	Yes
10	Yes	Yes
11	Yes	Yes

A number of studies have detailed mechanisms of torture and their specific pain sequalae. For example, falanga, or blunt trauma to the soles of the feet, may result in compensated gait and peripheral neuropathy, hanging from the limbs is associated with brachial plexopathy, and leg suspension or hyperextension is correlated with lumbosacral plexus injury [8]. Studies of asylum seekers have found that physical symptoms are approximately twice as frequent as psychological symptoms, and are two to three times as frequent in survivors of torture as compared to non-tortured asylum seekers. Psychiatric syndromes such as PTSD, Major Depressive Disorder, and somatization may contribute to chronic pain. Yet several studies of survivors demonstrate that the physical sequalae of torture actually accentuate or modulate the psychological sequelae, potentially more than the converse. Notably, the presence of chronic pain impedes psychiatric treatment; without diagnosing and treating pain in survivors of torture, both physical and psychiatric rehabilitation are incomplete and inadequate [9].

The United Nations’ Istanbul Protocol is the international standard for the medical evaluation of refugees [10]. It recommends a broad assessment of pain incorporated into the physical examination of individuals. Yet, physicians are not adequately performing this pain assessment in their evaluations of refugees [3]. Limitations potentially include the unfamiliarity of general physicians in diagnosing pain syndromes and the absence of a rapid screening tool for use, similar to the screening tools used for PTSD and MDD, which trigger evaluation by a specialist. Often, the complex clinical picture of survivors of torture results in the confounding or eclipsing of somatic pain by mental illnesses. This is noted to be particularly pronounced in developed countries where the clinical and research focus has been on the psychological rather than the physical impact of torture. The missed pain diagnoses impact treatment, as emphasis is placed on psychotherapeutic models that attribute pain to somatization, and primarily focus on psychological and alternate therapies during rehabilitation.

With the overemphasis on the psychological components of refugee health, the implementation of a rapid, chronic pain-screening tool for general providers in refugee clinics should be considered. The use of such a tool may trigger further questioning on pain or referral to a specialist. In this context, pain physicians, who have yet as a specialty to engage with refugee health, have a noteworthy diagnostic and treatment role to play. For example, Complex Regional Pain Syndrome, a devastating medical condition that may result from torture, is not mentioned in the Istanbul Protocol or any of the existing literature to-date on torture and pain, and would likely only be diagnosed and adequately managed by a pain specialist physician.

**Figure:**
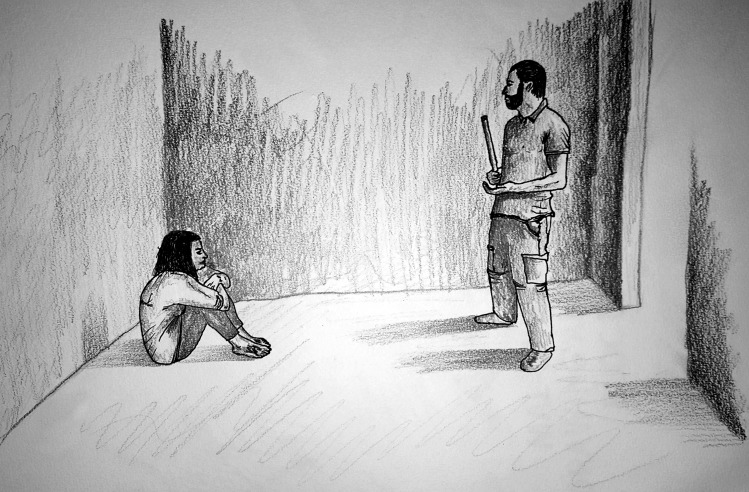
Photo: Image courtesy of ©Amnesty International / Mohamad Hamdoun (used with permission).

Future studies should aim to identify and validate an appropriate, rapid screening tool for pain to be used in refugee clinics. Guidance should be sought from experts in the field of refugee health, including general physicians, psychiatrists, medical anthropologists, and social workers, amongst others. Ultimately, pain specialist physicians must become integral to the medical care and rehabilitation of survivors of torture.
